# Folic Acid Supplementation Delays Atherosclerotic Lesion Development by Modulating MCP1 and VEGF DNA Methylation Levels In Vivo and In Vitro

**DOI:** 10.3390/ijms18050990

**Published:** 2017-05-05

**Authors:** Shanshan Cui, Wen Li, Xin Lv, Pengyan Wang, Yuxia Gao, Guowei Huang

**Affiliations:** 1 Department of Nutrition and Food Science, School of Public Health, Tianjin Medical University, 22 Qixiangtai Road, Heping District, Tianjin 300070, China; cuishanshan@tmu.edu.cn (S.C.); liwen828@163.com (W.L.); sailornju@hotmail.com (X.L.); wangpengyantj@163.com (P.W.); 2 Department of Cardiology, General Hospital of Tianjin Medical University, Tianjin 300052, China

**Keywords:** folic acid, DNA methylation, atherosclerosis, vascular endothelial growth factor, monocyte chemoattractant protein-1

## Abstract

The pathogenesis of atherosclerosis has been partly acknowledged to result from aberrant epigenetic mechanisms. Accordingly, low folate levels are considered to be a contributing factor to promoting vascular disease because of deregulation of DNA methylation. We hypothesized that increasing the levels of folic acid may act via an epigenetic gene silencing mechanism to ameliorate atherosclerosis. Here, we investigated the atheroprotective effects of folic acid and the resultant methylation status in high-fat diet-fed ApoE knockout mice and in oxidized low-density lipoprotein-treated human umbilical vein endothelial cells. We analyzed atherosclerotic lesion histology, folate concentration, homocysteine concentration, *S*-adenosylmethionine (SAM) and *S*-adenosylhomocysteine (SAH), and DNA methyltransferase activity, as well as monocyte chemotactic protein-1 (MCP1) and vascular endothelial growth factor (VEGF) expression and promoter methylation. Folic acid reduced atherosclerotic lesion size in ApoE knockout mice. The underlying folic acid protective mechanism appears to operate through regulating the normal homocysteine state, upregulating the SAM: SAH ratio, elevating DNA methyltransferase activity and expression, altering MCP1 and VEGF promoter methylation, and inhibiting MCP1 and VEGF expression. We conclude that folic acid supplementation effectively prevented atherosclerosis by modifying DNA methylation through the methionine cycle, improving DNA methyltransferase activity and expression, and thus changing the expression of atherosclerosis-related genes.

## 1. Introduction

Cardiovascular diseases (CVDs) are a major cause of morbidity and mortality in developed and developing countries [1]. Atherosclerosis (AS), a complex multifactorial cardiovascular disease, is an inflammatory disease characterized by initial lipid deposition [1,2]. When endothelial cells are damaged, various cytokines produced by the arterial wall participate in the initiation and progression of an inflammatory reaction. Thus, endothelial cell dysfunction is considered to be a key process in promoting atherosclerosis [3,4].

Recently, various studies have analyzed the involvement of epigenetic mechanisms in the development and progression of CVDs. Furthermore, a DNA methylation map of human AS has revealed differentially methylated Cytosine-phosphate-guanines (CpGs) are associated with AS onset as well as with endothelial and smooth muscle function [5,6,7]. The majority of DNA methylation occurs through DNA methyltransferases (DNMTs) which catalyze the addition of a methyl group to the C5 position of cytosine residues [8]. DNA methylation in promoter regions is associated with changes in gene expression and silencing and therefore aberrant DNA methylation may underlie CVD pathogenesis [9,10].

Epigenetic regulation provides a potentially reversible link among nutrition, one-carbon metabolism, and disease progression. A dietary deficiency of folic acid, an important component of transmethylation micronutrients, has been linked to endothelial dysfunction and CVDs including AS, coronary heart disease (CHD), anemia, and stroke via epigenetic changes [11,12,13]. The mechanism by which folic acid intake may modulate DNA methylation depends on the activity of specific enzymes, many of which are involved in the process of one carbon (1C) metabolism [14]. In particular, homocysteine (HCY), *S*-adenosylmethionine (SAM) (the universal methyl donor and central regulator of 1C fluxes), and *S*-adenosylhomocysteine (SAH) (considered to be a key toxic metabolite owing to its role as a methylation inhibitor) constitute key components of 1C metabolism [15]. The folate-dependent epigenetic mechanisms described above may thus be relevant to clinical CVDs [16].

However, the mechanism of the effect of folic acid supplementation on DNA methylation in the atherosclerotic process remains unclear. In the present study, we hypothesized that folic acid functions through an epigenetic gene silencing mechanism to lower AS-related gene expression in ApoE knockout (ApoE^−/−^) mice and human umbilical vein endothelial cells (HUVECs).

## 2. Results

### 2.1. Folic Acid Attenuates the Development of Atherosclerotic Lesions in ApoE^−/−^ Mice

To determine whether folic acid ameliorates the progression of atherosclerosis, we assessed lipid deposition in the aortic sinus of mice using Oil-Red O staining and stained with hematoxylin-eosin (HE) for morphology of the atherosclerotic lesions in aorta arch. The percentage of aortic luminal area covered by atherosclerotic lesions was quantified.

**Figure 1:**
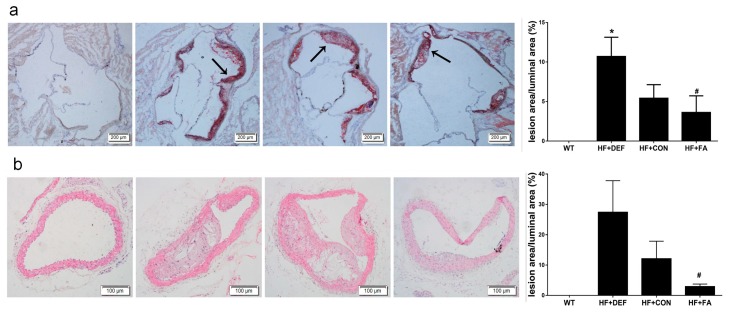
Intervention of folic acid ameliorated morphology of the atherosclerotic lesions in mice. Cryosections of the aortic sinuses were staining with Oil-Red O. Aortic arch was stained with hematoxylin and eosin (HE). (**a**) Representative images of aortic sinuses were stained with Oil-Red O (magnification ×40) for lipid deposition (red) and cross-stained with hematoxylin (blue). Lipid depositions were indicated with arrows. Quantitative analysis of the atherosclerotic lesions in aortic sinuses of mice; (**b**) Representative images of aortic arch were stained with HE (magnification ×100). Quantitative analysis of the atherosclerotic lesions in aortic arch of mice. The lesion area was measured as the percentage of luminal area covered by atherosclerotic lesions. Results are the mean ± SD. (*n* = 5 mice/group). *, *p* < 0.05 compared with the HF+CON group. #, *p* < 0.05 compared with the HF+DEF group.

Representative microscopy images of aortic sinus are shown in Figure 1a. Aortas from the wild-type (WT) group had very little lipid deposition. Conversely, there were extensive atherosclerotic lesions in the aortic sinus in mice on a high fat diet (HF+CON group), with the percentage of aortic cross-sectional luminal area occupied by Oil-Red O-stained lipid deposits being increased in ApoE^−/−^ mice. These results suggested that we had successfully established this mouse atherosclerotic model. Folic acid deficiency (HF+DEF group) significantly aggravated the extent of atherosclerotic lesions at the aortic sinus, as evidenced by the increased percentage of aortic cross-sectional luminal area occupied by the stain (*p* < 0.05 versus HF+CON group, Figure 1b). In contrast, the addition of folic acid to the atherogenic diet (HF+FA group) inhibited the development of atherosclerotic lesions and decreased the percentage of aortic cross-sectional luminal area occupied by the stain (5.41 ± 1.72% and 3.62 ± 2.08%, respectively).

At 24 weeks of age, apparent atherosclerotic lesions were observed in cross-section of ApoE^−/−^ mice, but not in the WT controls, only fatty streaks were observed (Figure 1b). The percentage of surface lesion was increased almost 2.2-fold in HF+DEF group compared with HF+CON group. Compared with the folic acid deficient group, the percentage of lumen areas was upregulated significantly with folic acid supplementation. (*p* < 0.05, 27.4 ± 10.4% and 2.9 ± 1.9%, respectively) (Figure 1b). These results indicated that the addition of folic acid was anti-atherogenic in ApoE^−/−^ mice.

### 2.2. Supplementation with Folic Acid Raises the Folate Concentration and Decreases Homocysteine Concentration In Vivo and In Vitro

**Figure 2:**
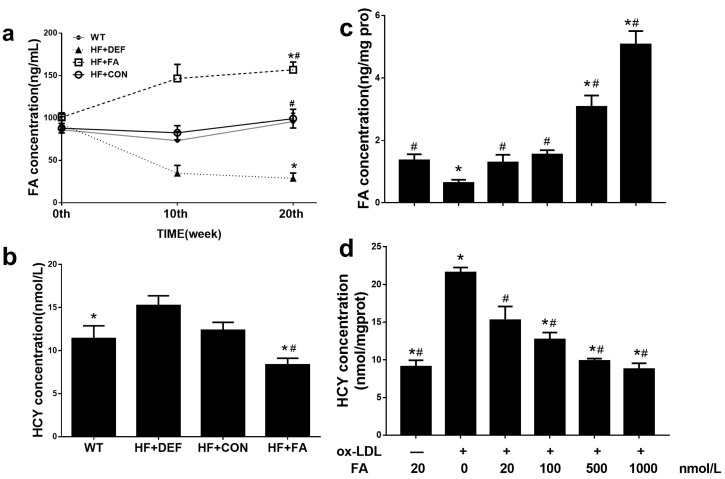
Responses of folate concentration and homocysteine concentration in apolipoprotein E-deficient (ApoE^−/−^) mice and human umbilical vein endothelial cells (HUVCEs). (**a**) Time course of serum folate concentration changes during the 20 experimental weeks among all of the study groups fed folate-deficient diet and folic acid supplements; (**b**) Serum homocysteine concentration in which ApoE^−/−^ mice were treated for 20 experimental weeks. Results are the mean ± SD. (*n* = 8 mice/group). *, *p* < 0.05 compared with the HF+CON group. #, *p* < 0.05 compared with the HF+DEF group; (**c**) Folic acid increased intracellular folate concentration in a dose-dependent manner in HUVCEs treated with oxidized low-density lipoprotein (Ox-LDL); (**d**) Folic acid decreased intracellular homocysteine concentration in a dose-dependent manner in HUVCEs. The plotted values are mean ± SD values for 3 experiments. *, *p* < 0.05 compared with the Ox-LDL+FA20. #, *p* < 0.05 compared with the Ox-LDL+FA0.

All groups of mice had similar levels of serum folate prior to the various interventions. After 10 weeks, the serum folate concentration was 57.9% lower in the folate-deficient diet group compared to the control group. In contrast, folic acid supplementation by gavage raised the serum folate concentration by 77.5% (Figure 2a) compared to the control group. Subsequently, we measured the serum folate level changes from 10 to 20 weeks and found that the concentrations were maintained on three different levels. After 20 weeks, animals on the folic acid-deficient diet had the lowest level of serum folate concentration (28.8 ± 16.5 ng/mL). Compared with HF+CON, folic acid supplementation, via folic acid gavage, significantly raised the serum folate concentration and decreased the plasma HCY concentration (*p* < 0.05; Figure 2a,b), confirming that folic acid supplementation increased serum folate levels and decreased plasma HCY levels in mice.

In HUVECs, compared with the oxidized low-density lipoprotein (Ox-LDL+FA20 group) alone group, incubation with folic acid increased the intracellular folate concentration in a dose-dependent manner and this effect was significant at folic acid levels of 500–1000 nmol/L (*p* < 0.05, Figure 2c). Additionally, exposure to higher concentrations of folic acid (500–100 nmol/L) reduced the intracellular HCY concentration compared to the Ox-LDL+FA20 group (*p* < 0.05, Figure 2d). These results indicated that the addition of folic acid attenuated the HCY concentration in HUVECs treated with Ox-LDL.

### 2.3. Folic Acid Does Not Alter Body Weight or the Amount of Food Intake in ApoE^−/−^ Mice, but Reduces the Concentration of Serum Total Cholesterol

Compared with WT mice, in ApoE^−/−^ mice, a significant increase in body mass was observed in the HF+DEF, HF+CON, and HF+FA treatment groups. The increases in weight were all attributable to the high fat diet and were not due to folic acid (Table 1). There was no significant change in food intake among all of the treatment groups. The average food-intakes for the WT, HF+DEF, HF+CON, and HF+FA groups were 3.97 ± 0.44, 4.29 ± 0.17, 3.65 ± 0.07, and 4.40 ± 0.58 g, respectively.

ApoE^−/−^ mice also developed severe hypercholesterolemia, showing an almost 3.8-fold increase in total cholesterol (TC) compared with WT controls of the same age (Table 1). Folic acid supplementation significantly reduced TC levels (*p* < 0.05) (Table 1). In contrast, triglyceride (TG) concentrations were not generally affected by folic acid treatment.

### 2.4. Folic Acid Increases DNMT Expression, Activity, and Methylation Potential

**Figure 3:**
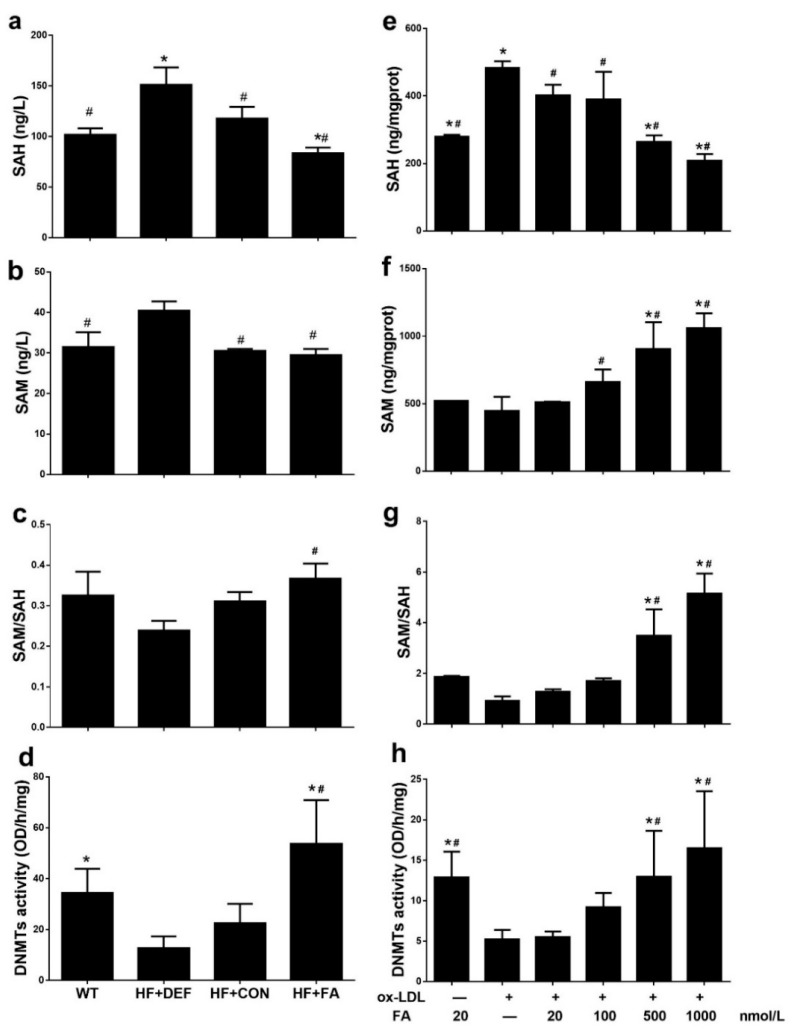
Responses of DNA methyltransferase (DNMT) activity and methylation potential in ApoE^−/−^ mice and HUVCEs. The ApoE^−/−^ mice fed a WT, HF+DEF, HF+CON and HF+FA diet during the 20 experimental weeks. HUVCE were treated with Ox-LDL for 24 h and folic acid in 48 h. (**a**) *S*-adenosylhomocysteine (SAH) concentration in blood plasma; (**b**) *S*-adenosylmethionine (SAM) concentration in blood plasma; (**c**) Methylation potential (SAM: SAH ratio) in blood plasma; (**d**) The DNMTs activity of aortic. Results are the mean ± SD. (*n* = 5 mice/group). *, *p* < 0.05 compared with the HF+CON. #, *p* < 0.05 compared with the HF+DEF; (**e**) SAM concentration in HUVECs; (**f**) SAH concentration in HUVECs; (**g**) Methylation potential (SAM: SAH ratio) in HUVECs; (**h**) The total DNMTs activity in cells. The plotted values are mean ± SD values for 3 experiments. *, *p* < 0.05 compared with the Ox-LDL+FA20. #, *p* < 0.05 compared with the Ox-LDL+FA0.

SAM and SAH concentrations were measured in mouse plasma from the in vivo study and in HUVECs from the in vitro study. In vivo, compared to HF+CON, folate deficiency increased plasma SAH and SAM concentrations by 28.3% and 32.3%, respectively, while decreasing the methylation potential (expressed as the SAM: SAH ratio) by 23.0% (Figure 3a–c). In the HUVECs, folic acid dose-dependently increased the SAM concentration and lowered the SAH concentration, thereby increasing the methylation potential (Figure 3e–g). In particular, the highest methylation potential was achieved with 1000 nmol/L folic acid (5.142 ± 0.7969; Figure 3g). Folic acid also increased the methylation potential in ApoE^−/−^ mice fed with a high-fat diet and in HUVECs treated with Ox-LDL.

To determine global DNMT activity levels, we prepared and assayed fresh nuclear extracts. The results shown in Figure 3d indicate that DNMT activity was inhibited in the ApoE^−/−^ atherosclerotic model, compared with WT mice (22.46 ± 7.618 versus 34.33 ± 9.547 OD/h/mg, respectively). However, DNMT activity was significantly upregulated in HF+FA mice (*p* < 0.05 versus HF+CON). In addition, exposure to Ox-LDL decreased DNMT activity in HUVECs incubated with 20 nmol/L folic acid compared with those cells cultured under normal conditions (*p* < 0.05), whereas DNMT activity was fully protected by increasing the folic acid concentration. Higher folic acid concentrations (500–1000 nmol/L) significantly increased DNMT activity compared to the Ox-LDL+FA20 group (*p* < 0.05), with highest DNMT activity (16.49 ± 7.043 OD/h/mg) being observed at a folic acid concentration of 1000 nmol/L (Figure 3h). Folic acid thus enhanced DNMT activity in ApoE^−/−^ mice fed a high-fat diet and in HUVECs treated with Ox-LDL.

**Figure 4:**
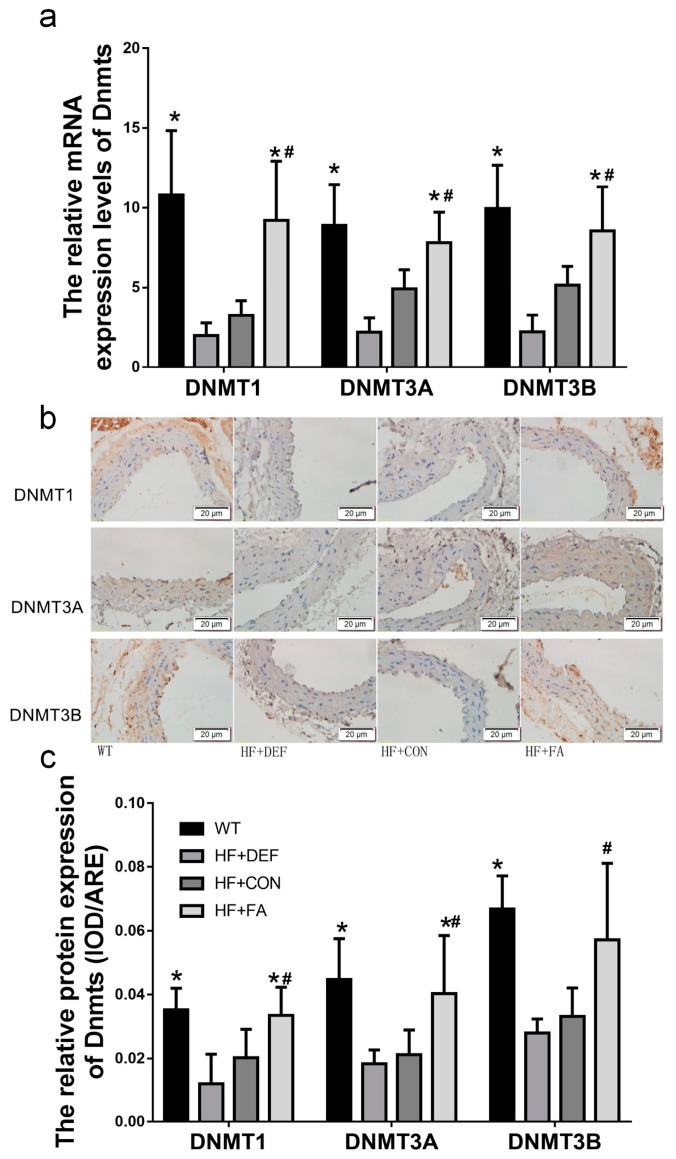
Folic acid modulates the expression of DNMT1, DNMT3A, and DNMT3B in mice. The ApoE^−/−^ mice fed a WT, HF+DEF, HF+CON and HF+FA diet during the 20 experimental weeks. (**a**) Representative images of aortic cross-sections immunostained with markers of DNMT1, DNMT3A, and DNMT3B, cell nuclei were stained with Mayer’s hemalaun solution (blue); (**b**) Summary of DNMT1, DNMT3A, and DNMT3B protein expression levels shows mean ± SD. for IOD of immunoreactive DNMT1, DNMT3A, and DNMT3B (*n* = 5 mice/group); (**c**) Gene expression levels of DNMT1 (×10^2^), DNMT3A (×10^5^), and DNMT3B (×10^5^) in aortic of ApoE^−/−^ mice (*n* = 5 mice/group). *, *p* < 0.05 compared with the HF+CON group. #, *p* < 0.05 compared with the HF+DEF group.

We next examined DNMT1, DNMT3A, and DNMT3B expression in mice. First, the expression of individual DNMT isoforms was determined using quantitative real-time polymerase chain reaction (PCR) analysis. Figure 4a shows that folic acid gavage significantly increased the mRNA expression of DNMT1, DNMT3A, and DNMT3B (*p* < 0.05 versus HF+CON). In contrast, the levels of DNMT1, DNMT3A, and DNMT3B mRNA expression were downregulated in the folate-deficient diet relative to the HF+CON group (39.1%, 55.4%, and 57.1%, respectively). The respective DNMT isoform protein levels were also determined using an immunohistochemical analysis of the aortic arch. Figure 4c shows that the levels of DNMT1, DNMT3A, and DNMT3B proteins were downgraded in the folate-deficient diet group. Conversely, folic acid gavage significantly increased the protein expression of DNMT1 and DNMT3A (*p* < 0.05 versus HF+CON). Thus, folic acid increased DNMT expression in mice fed a high-fat diet.

### 2.5. Folic Acid Ameliorates MCP1 and VEGF Methylation Levels and Expression In Vivo and In Vitro

**Figure 5:**
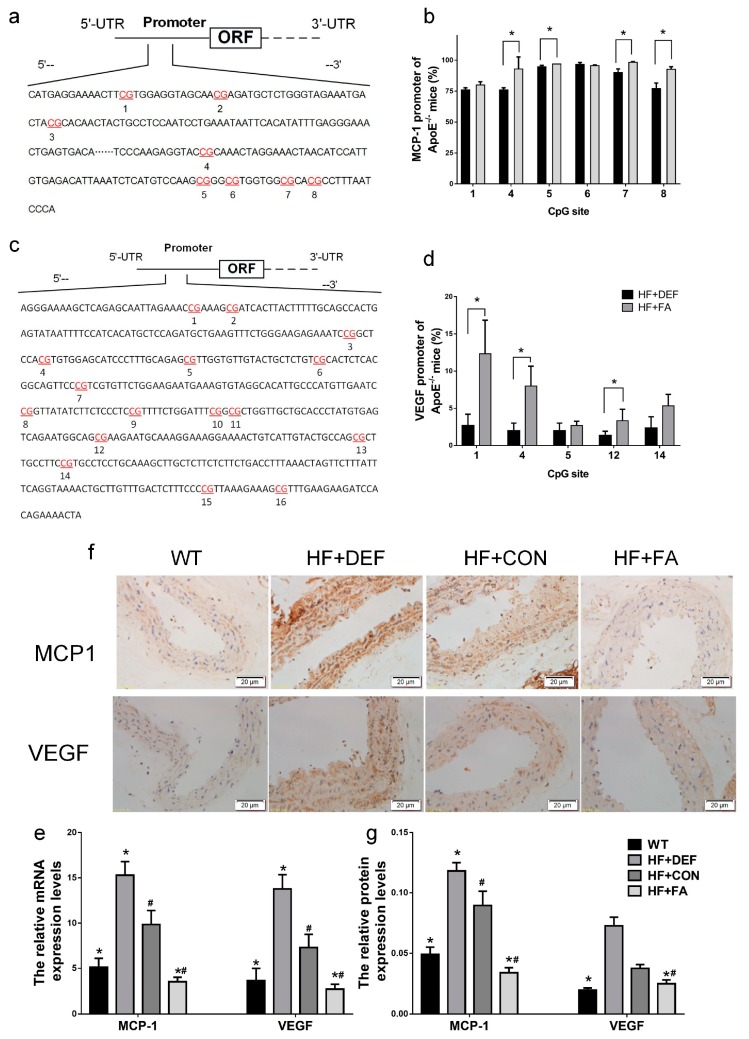
Folic acid modulates the monocyte chemotactic protein-1 (MCP1) and vascular endothelial growth factor (VEGF) promoter methylation levels of the CpG sites and the expression of MCP1 and VEGF in mice. The ApoE^−/−^ mice fed a WT, HF+DEF, HF+CON and HF+FA diet during the 20 experimental weeks. (**a**) Schematic diagram of mice MCP1 promoter (68 to 421 bp). Pyrosequencing assay data were evaluated by dividing the MCP1 promoters into 8 CpG sites. The locations of CpG sites were indicated with red font numbers; (**b**) Mean methylation levels of CpG sites in MCP1 in aorta of ApoE^−/−^ mice (*n* = 3 mice/group). *, *p* < 0.05 compared with the HF+DEF; (**c**) Schematic diagram of mice VEGF promoter (1930 to 2372 bp). Pyrosequencing assay data were evaluated by dividing the VEGF promoters into 16 CpG sites. The locations of CpG sites were indicated with red font numbers; (**d**) Mean methylation levels of CpG sites in VEGF in aorta of ApoE^−/−^ mice (*n* = 3 mice/group). *, *p* < 0.05 compared with the HF+DEF; (**e**) Gene expression levels of *MCP1* and *VEGF* in aortic of ApoE^−/−^ mice were analyzed by real-time PCR (MCP1 ×100; VEGF ×1000). The mRNA of β-actin was quantified as an endogenous control; (**f**) Representative images of aortic cross-sections immunostained with markers of MCP1 and VEGF, cell nuclei were stained with Mayer’s hemalaun solution (blue); (**g**) Summary of protein expression levels for IOD of immunoreactive MCP1 and VEGF. Results are the mean ± SD. (*n* = 5 mice/group). *, *p* < 0.05 compared with the HF+CON. #, *p* < 0.05 compared with the HF+DEF.

We next examined monocyte chemotactic protein-1 (MCP1) and vascular endothelial growth factor (VEGF) promoter demethylation in the mouse aorta to determine whether the expression of these genes is regulated by methylation. The DNA fragments from the MCP1 and VEGF proximal promoters were divided into 8 and 16 CpG sites, respectively (Figure 5a,c). The methylation levels at the different CpG sites were evaluated using a pyrosequencing assay. Figure 5b and d show that folic acid supplementation significantly raised the methylation percentage across multiple CpG sites. Specifically, methylation was significantly upregulated by folic acid supplementation at CpG sites 4, 5, 7, and 8 in the MCP1 promoter (*p* < 0.05 versus HF+DEF, Figure 5b). Folic acid also increased DNA methylation levels at CpG sites 1, 4, and 12 in the VEGF promoter (*p* < 0.05 versus HF+DEF, Figure 5d). For the VEGF promoter region, the highest methylation level (12.3 ± 4.5%) was found at the first CpG site. Folic acid, therefore, modulated methylation patterns in the MCP1 and VEGF promoters in the ApoE^−/−^ mice aorta.

MCP1 and VEGF gene and protein expression in the ApoE^−/−^ mice aorta were next evaluated using real-time PCR and immunohistochemical analysis. Figure 5 shows that the expression of MCP1 and VEGF mRNA and protein levels were significantly increased in ApoE^−/−^ mice compared to WT mice (*p* < 0.05). Conversely, folic acid gavage significantly decreased the expression of these mRNAs and proteins (*p* < 0.05 comparing HF+CON to HF+FA). Thus, folic acid inhibited the expression of MCP1 and VEGF mRNA and protein in ApoE^−/−^ mice fed a high-fat diet.

## 3. Discussion

The present study established that gene methylation links nutrition to endothelial function in experimental models of AS using folic acid supplementation as an intervention in ApoE^−/−^ mice and HUVECs exposed to Ox-LDL. Our data demonstrated that folic acid ameliorated atherosclerosis in ApoE^−/−^ mice and also altered the abundance of MCP1 and VEGF expression. This effect of folic acid was associated with increased methylation potential and DNMT activity, as well as with altered DNA methylation at the MCP1 and VEGF promoters.

In countries where folic acid is added to staple foods, a decline in cardiovascular risk, especially stroke, has been noted [17,18]. These observations have opened a new area of research investigating the association of folic acid with the prevention of cardiovascular diseases. Observational studies have indeed suggested that folic acid supplementation may reduce the progression of early stage, sub-clinical, atherosclerosis and future stroke risk in healthy elderly people, and in people not residing in regions with mandatory grain fortification, who are more likely to be deficient in B vitamins [12,19,20,21]. However, the relationship between folic acid and the reduced risk of cardiovascular disease in the general healthy population is controversial [22,23]. An alternative possibility is that folic acid reducing homocysteine may be beneficial only at early stages of vascular disease, but less effective in the face of late-stage atherosclerosis. In the current study, folic acid deficiency aggravated atherosclerotic lesion development, and folic acid supplementation partly ameliorated vascular endothelial dysfunction both in vitro and in vivo. Our results indicate that folic acid may partly ameliorate the higher risk of developing cardiovascular disease. These results may be beneficial to the further research of the anti-inflammatory effects of folic acid. Atherosclerosis is a chronic inflammatory process, and this process is mediated by chemotactic factors [24,25]. Previous studies indicated that folic acid was also found to exert anti-inflammatory effects in macrophages [26,27]. These studies suggested future studies will need to focus on the mechanisms underlying these anti-inflammatory effects in macrophages and the signal transduction pathway affected by folic acid.

AS constitutes a chronic disease of the arterial wall that has been established as a complex inflammatory disease [3]. MCP1 and VEGF act as basic mediators in a biological system that plays a significant role in the early stages of AS formation [28,29]. A related study also suggested that ApoE^−/−^ mice with increased MCP1 secretion were at an increased risk of AS [30]. VEGF, a potent growth factor for endothelial cells and inducer of angiogenesis, is not only important for endothelial integrity and thus for vascular function, but may also enhance the pathophysiologic mechanism of plaque formation [31]. Thus, we investigated whether folic acid affected these two factors. After 20 weeks of treatment, folic acid supplementation decreased both aortic MCP1 and VEGF levels in ApoE^−/−^ mice. Combined with the histologic evaluations of atherosclerotic lesions, these data suggest that folic acid attenuated atherosclerotic lesions by decreasing the expression of inflammatory factors and improving vascular endothelial cell function.

Environmental factors that induce methylation of gene promoters may regulate gene expression, because diets deficient in trans-methylation micronutrients (such as folate) may cause hypomethylation of promoter regions in AS-relevant genes [13,32,33]. In turn, aberrant epigenetic control at CpG-islands may contribute to AS pathology [34]. Studies have indicated that MCP1 promoter DNA hypomethylation may play a key role in the formation of AS in hyperhomocysteinemia [9]. However, the study of aberrant DNA methylation in AS is at an early stage and only a very small number of individual genes has been examined. In the current study, the MCP1 and VEGF promoters were found to be hypomethylated and the level of MCP1 hypomethylation was found to highest in the HF+FA group. The current prevailing explanation for folate-induced DNA hypomethylation is that SAM functions as the methyl donor for DNA methylation modification and is central to the regulation of many biological processes. Gene hypomethylation may therefore be due to a depletion of SAM and an elevation of intracellular SAH (i.e., an increase in the SAM:SAH ratio), resulting in an overall decrease in methylation potential [35]. The accumulation of SAH can affect DNA methylation patterns by causing a feedback inhibition of SAM-dependent methyltransferases [36]. The present findings support the hypothesis that inhibition of AS by folic acid is mediated by an epigenetic mechanism because the DNA methylation rate increased with the amount of folic acid ingested in vivo from a gavage or applied in vitro through a culture medium. Folic acid induced increases in DNA methylation rates would be expected to silence MCP1 and VEGF proteins in both mice and HUVECs. In addition, folic acid reduced SAH level in vivo and in vitro. It has been reported that plasma SAH is a more sensitive biomarker of cardiovascular disease than homocysteine and that SAH represents a critical pathological factor in homocysteine-associated disorders [37]. Therefore, we hypothesized that folic acid may also participate in AS-related protein promoter DNA hypomethylation via the methionine cycle.

DNA methylation, i.e., the addition of methyl groups to a cytosine base at CpG dinucleotide residues, is catalyzed by DNMTs. The degree of DNA methylation regulates gene expression patterns by altering chromatin structure. Among the three active DNMTs that have been identified in mammals, DNMT3A and DNMT3B are primarily responsible for de novo methylation in embryonic and postnatal tissues, whereas DNMT1 subsequently maintains the methylated state [38]. The expression of these methyltransferases was found to be increased in mice fed folic acid supplements as compared with the mice in the folate-deficient diet group. In addition, folic acid induced the expression of functional DNMT isoforms, because it increased DNMT activity in nuclear extracts. Consequently, folate deficiency leads to depletion of the methyl pool with subsequent under-methylation of critical genes, as has been previously reported [35]. It has also been shown that DNMT activity may be affected by SAM/SAH levels in cells, as SAH is a reversible DNMT inhibitor [39,40]. Taken together, these findings are consistent with a mechanism by which folic acid inhibits AS through increases in methylation potential, DNMT activity, the DNA methylation rate at the MCP1 and VEGF promoters, and gene silencing of MCP1 and VEGF.

Folate is necessary for cell growth. In the current study, HUVECs were cultured in vitro under conditions designed to mirror those in vivo [12,19,41]. As M199 medium contains folic acid at 20 nmol/L as a standard constituent, we therefore used HUVEC cells grown in this medium as the control group to represent the baseline condition. We considered 100 nmol/L folic acid as a low-dose supplement concentration in vitro, to be representative of human serum folate concentrations after folic acid supplementation [12]. Consequently, we considered 500 and 1000 nmol/L folic acid (i.e., five- and ten-fold more concentrated than the low-dose supplement concentration), respectively, as medium- and high-dose supplement concentrations in vitro. HUVECs were exposed to these concentrations of folic acid for 48 h to study the effect of folic acid on the acute toxicity of Ox-LDL in HUVECs.

A limitation of the present study is the use of solely male mice to study the effect of folic acid on atherosclerosis. However, this study mainly focused on the inhibition of folic acid on atherosclerosis such that the addition of female mice would result in a sex effect mediated by estrogens therefore potentially undermining our conclusion. Epidemiological studies [42,43,44] have shown that age and sex clearly affect the pathogenesis of atherosclerosis in humans. Until menopause, females are relatively protected from cardiovascular disease, presumably due to an effect of estrogen [45]. In experimental atherosclerotic models, especially in mice fed a high-fat diet, it is known that estrogens against LDL could be inhibit in atherogenesis [46,47,48]. For this reason, the present study used solely male mice to avoid confounding factors from estrogen as was shown in the Han JM et al. [49] and Peng J et al. [50] studies. Another limitation is that the experiment was conducted on mice and cells, the conclusions cannot be applied to humans in directly.

## 4. Materials and Methods

### 4.1. Animals, Diets, and Experimental Procedures

A total of 24 homozygous male ApoE^−/−^ mice on a C57BL/6J background aged 4 weeks were purchased from Peking Huafukang Laboratory Animal Center (Beijing, China) and randomly distributed into three groups (8 per group): (1) high-fat + folic acid-deficient diet plus daily intragastric gavage with 0.9% saline (HF+DEF); (2) high-fat diet plus daily intragastric gavage with 0.9% saline (HF+CON); and (3) high-fat diet plus daily intragastric gavage with 60 μg/kg folic acid (HF+FA). In addition, 8 age-matched C57BL/6J mice as WT controls received high-fat diet plus daily intragastric gavage with 0.9% saline.

All mice were fed with a Western-type high-fat diet [51] (21% fat, 1.25% cholesterol) for 20 weeks. The folate-deficient diet (containing 0.2 mg folic acid/kg diet) and the control diet (2.1 mg folic acid/kg diet) were purchased from TestDiet (St. Louis, MO, USA). All mice were fed with specific diets and gave intragastrical administration at 4 weeks of age, and the treatments lasted for 20 weeks when the mice reached the age of 24 weeks. Water and food were provided ad libitum over the 20-week experimental period. The mice were individually housed in metabolic cages in a temperature-controlled room (22.5 ± 0.5 °C) with a 12-h light, 12-h dark cycle. The study was approved by the ethics committee of Tianjin Medical University (TMUaMEC 2015009).

After 20 weeks, the mice (24 weeks of age) were fasted overnight (12 h) and then sacrificed via suffocation with CO_2_ and euthanized by extracting blood by cardiac puncture with a syringe. The blood was collected and centrifuged for the preparation of plasma and serum. The plasma, serum, heart, and aorta were immediately collected and stored at −80 °C until analysis.

### 4.2. HUVECs Culture

HUVECs were obtained from Guangzhou Jennio Biotech Co., Ltd. (Guangzhou, China). HUVECs were cultured in M199 medium with 10% (*v*/*v*) fetal bovine serum (Gibco BRL, Grand Island, NY, USA), 100 IU/mL penicillin G, and 100 IU/mL streptomycin. HUVECs were maintained in a humidified atmosphere of 5% CO_2_ in an incubator at 37 °C. HUVECs were used at passages 3–5.

All the HUVECs in the treatment groups were exposed to medium containing 120 μg/mL of Ox-LDL for 24 h and then exposed to the indicated concentrations of folic acid (0–1000 nmol/L) for 24 h. These HUVECs were assigned to five treatment groups: (1) folate-deficient M199 medium (FA0); (2) folic acid-free M199 medium (FA20); (3) folic acid-free M199 medium plus low folic acid (100 nmol/L) (FA100); (4) M199 medium plus medium folic acid (500 nmol/L) (FA500); and (5) M199 medium plus high folic acid (1000 nmol/L) (FA1000). HUVECs were also incubated with folic acid-free M199 medium as WT control (WT).

### 4.3. Histomorphometric Analysis of Plaque Morphology

Oil red O staining was used to assess lipid content. Hematoxylin-eosin (HE) staining was used to assess pathological changes.

The heart samples were embedded in tissue freezing medium optimum cutting temperature compound and the aortic sinus was sectioned into consecutive 8-μm thick sections at −20 °C. The distal end of the aortic sinus was identified by the disappearance of the three aortic valve cusps as previously described [52]. For quantitative analysis of AS, every sixth section (spacing 50 μm apart) from each mouse was stained with Oil Red O and observed under 40× magnification (Olympus IX81, Tokyo, Japan).

The aorta (from aortic opening to 1 cm from the opening section of the aorta) was fixed in 10% neutral buffered formalin, dehydrated in a graded series of ethanol, and embedded in paraffin. Thereafter, paraffin block samples were sectioned to achieve a thickness of 5 μm and stained with hematoxylin-eosin (HE) for histological examination. HE-stained slides were observed under 100× magnification (Olympus IX81).

Atherosclerotic lesions were quantitatively analyzed using Image-Pro Plus (IPP) software (version 6.0, Media Cybernetics, Inc., Silver Spring, MD, USA). The lesion area index was calculated as the percentage of the aortic lumen area covered by atherosclerotic lesions.

### 4.4. Assessment of Folate Levels in Serum and Cells

Folate levels were measured using a competitive protein-binding assay with an automated chemiluminescence immunoassay analyzer (IMMULITE 2000 XPi, Siemens Healthcare Diagnostics Inc., Malvern, PA, USA) according to the manufacturer's specifications. High and low folate standards provided in the kit were used to correct the automated chemiluminescent system; the detectable concentration ranged from 1–24 ng/mL. As the serum folate levels were high, samples were diluted five times with 0.9% saline. This system was able to detect folic acid, dihydrofolate, and tetrahydrofolate. Folate levels in cells were normalized to protein content, as determined by the BCA Protein Assay Kit (BosterBio, Wuhan, China).

### 4.5. Assessment of HCY Levels in Serum and Cells

Plasma HCY levels were measured using a colorimetric enzymatic kit (Ningbo Meikang Biological Technology Company, Ningbo, China) according to Richmond’s colorimetric procedure and Trinder’s colorimetric method. The assays were performed using an automatic biochemical analyzer DIRUI CS-T300 (Dirui Medical Polytron Technologies Inc., Changchun, China). In addition, samples were diluted in 0.9% saline to allow for the fact that the examination area of this system spanned only 3.0–50 μmol/L.

### 4.6. DNMT Activity Assay

Nuclear extracts of stored frozen aorta tissue and HUVECs were isolated using the nuclear extraction kit (Epigentek Group Inc., Farmingdale, NY, USA). DNMT activity was measured using a DNMT activity/inhibition assay kit (Colorimetric) according to the manufacturer’s instructions (Epigentek Group Inc., Farmingdale, NY, USA). A lot-specific standard curve was created using the DNMT1 provided in the kit. Optical density (OD) was measured on a microplate reader at 450 nm and DNMT activity (OD/(h·mg)) was calculated according to the following formula:

$$\mathrm{DNMT}\;\mathrm{activity}(\mathrm{OD}/h/\mathrm{mg})=\frac{\mathrm{average}\;\mathrm{sample}\;\mathrm{OD}\;-\;\mathrm{average}\;\mathrm{blank}\;\mathrm{OD}}{\mathrm{proteinamount}(\mu g)*\times \mathrm{hour}**}\times 1000$$

where * indicates protein amount added into the reaction and ** indicates incubation time used for the reaction.

### 4.7. Methylation Potential Assay

As a pretreatment, HUVECs (2 × 10^7^) were washed twice with cold phosphate buffered saline (PBS). The cellular protein content was determined using the BCA protein assay kit (BosterBio, Wuhan, China). Subsequently, the HUVECs and plasma were resuspended ice-cold perchloric acid (0.4 mol/L) and centrifuged at 20,000× *g* for 10 min at 4 °C. The supernatants were collected and filtered through 0.45 μm membrane (Millipore, Billerica, MA, USA) prior to application to a high-performance liquid chromatography system (Waters, Milford, MA, USA) containing a Venusil MP-C18 column (250 mm × 4.6 mm, 5 μm particles; Agela Technologies, Wilmington, DE, USA) fitted with a matched guard column and an ultraviolet detector. The mobile phase contained 50 mmol/L sodium dihydrogen phosphate and 10 mmol/L sodium heptanesulfonate (pH 4). Elution of SAM and SAH was performed at a flow rate of 1 mL/min. Absorption of eluted compounds was monitored at λ = 254 nm. The SAM and SAH elution peaks were identified by comparing to SAM and SAH standards. Cellular SAM and SAH concentrations were normalized to cellular protein content.

### 4.8. Real-Time PCR Analysis

Gene expression was quantified by real-time reverse transcription-PCR. Total RNA was isolated from the stored frozen aorta tissue using the Eastep^®^ Super Total RNA Extraction Kit (Promega Corporation, Madison, WI, USA). First-strand cDNA was reverse transcribed from 2 μg total RNA using MMLV reverse transcriptase. The 20 μL reaction volume was incubated for 60 min at 42 °C and for 10 min at 70 °C before storage at −20 °C. Quantitative PCR was performed using the Roche LightCycler 480 sequence detector (Roche, Mannheim, Germany). The cDNA was amplified using a 20 μL PCR mixture including 10 μL LightCycler 480 SYBR Green I Master Kit (Roche), 5 μL cDNA, 1 μL forward primer, 1 μL reverse primer (Table 2), and 3 μL water (PCR grade). The reaction mixtures were incubated at 95 °C for 5 min, followed by 45 amplification cycles (denaturation, 95 °C for 10 s; annealing for 10 s, at 59 °C for DNMT1, DNMT3A, and at 63 °C for DNMT3B, MCP1, and VEGF; extension, 72 °C for 10 s). The specificity of each primer pair was confirmed by melting curve analysis and agarose-gel electrophoresis. Each sample was analyzed in duplicate and quantification was performed with the efficiency-corrected 2^−Δ^*^C^*^t^ method using housekeeping genes β-actin for internal normalization.

### 4.9. Immunohistochemistry

Aortic arch samples fixed in 4% paraformaldehyde, embedded in paraffin, and sectioned into consecutive 8 μm thick free-floating sections were incubated with 4% bovine serum albumin (BSA) in PBS for 1 h and then reacted with one of the following antibodies: a rabbit polyclonal anti-MCP1 antibody, 1:200 (Abcam, Cambridge, UK); a rabbit polyclonal anti-VEGF antibody, 1:200 (Abcam); a mouse monoclonal anti-DNMT1 antibody, 1:500 (Abcam); a rabbit polyclonal anti-DNMT3A antibody, 1:300 (Abcam); or a rabbit polyclonal anti-DNMT3B antibody, 1:200 (Abcam) at 4 °C overnight. The sections were washed with PBS and reacted with the appropriate biotinylated secondary antibodies, 1:1000 (Cell Signaling Technology, Danvers, MA, USA) in PBS and visualized using an SABC Elite kit (BosterBio Technology., LTD., Wuhan, China). Quantitation of immunoreactive protein was performed on a random five sections and the images were obtained using a microscope (Olympus). The integrated optical density of each was determined using IPP software (Media Cybernetics, Inc., Silver Spring, MD, USA).

### 4.10. MCP1 and VEGF Promoter Methylation Analyses

The quantitative methylation analysis of CpG sites of MCP1 and VEGF promoter regions were determined using the Sequenom MassARRAY platform (CapitalBio, Beijing, China). This system uses RNase-specific enzyme digestion in combination with matrix-assisted laser desorption/ionization time-of-flight (MALDI-TOF) mass spectrometry. Genomic DNA was extracted from aorta tissue using the QIAamp DNA Mini Kit (Qiagen, Dusseldorf, Germany) according to the manufacturer’s instructions. DNA concentration and purity were determined by electrophoresis on a 0.8% agarose gel and UV absorbance at 260 and 280 nm. A total of 1.5 μg genomic DNA from each sample was bisulfite-treated with the EZ DNA Methylation-Gold Kit (Zymo Research, Irvine, CA, USA) according to the manufacturer’s instructions. Real-time PCR was performed using the PCR Accessory Set (Sequenom, San Diego, CA, USA). PCR primers were designed using Epidesigner (available online: http://www.epidesigner.com). For each reverse primer, an additional T7 promoter tag for in vivo transcription was added, as well as a 10-mer tag on the forward primer to adjust for melting temperature differences. We used the following primers based on the reverse complementary strands of *MCP1* (5′-aggaagagagTTGTGGGTATTTATGAATATGAGGAA-3′ and 3′-cagtaatacgactcactatagggagaaggctAAATTTACCTACCTCTACCTCCCAA-5′) and *VEGF* (5′-aggaagagagTGGTTAGTTTTTTGTGGATTTTTTT-3′ and 3′-cagtaatacgactcactatagggagaaggctACAACCACTAAATATAATTTTCCATCA-5′). PCR products were purified and digested using the MassCLEAVE Kit (Sequenom). Mass spectra were obtained via Spectro CHIP^®^ Arrays and Clean Resin Kit (Sequenom) and MassARRAY Compact MALDI-TOF (Sequenom). The methylation ratios were generated and analyzed using EpiTYPER software (Sequenom).

### 4.11. Plasma Lipid Parameters Analysis

For determination of the lipid profile, blood samples were collected at baseline (4 weeks age) and the end of the diet treatment period (24 weeks age). Plasma total cholesterol (TC) and triglyceride (TG) levels were measured using a colorimetric enzymatic kit (Ningbo Meikang Biological Technology Company, Ningbo, China) in accordance with the Richmond’s colorimetric procedure and Trinder’s colorimetric method. The assays were performed using an automatic biochemical analyzer DIRUI CS-T300 (Changchun Dirui Medical Polytron Technologies Inc., Changchun, China).

### 4.12. Statistical Analysis

Data were expressed as the means ± SD and analyzed using the software package SPSS 16.0 (Chicago, IL, USA). One-way ANOVA and the Student-Newman-Keuls test for multiple comparisons were used to evaluate significant differences among the experimental groups. Student’s one-tailed *t*-tests were performed to evaluate the significance of any differences between the MALDI-TOF mass spectrometry data with the data from bisulfite sequencing between test groups. *p* < 0.05 was considered significant.

## 5. Conclusions

In conclusion, supplementation with folic acid increased the methylation potential and DNMT activity, modified DNA methylation, and ameliorated MCP1 and VEGF levels in ApoE^−/−^ mice and in HUVECs exposed to Ox-LDL. These findings are largely consistent with the theoretical mechanism of action of folic acid. This study may provide new insights regarding the use of folic acid as nutritional adjunct to prevent AS in high-risk individuals.

## Figures and Tables

**Table 1:** Mean body weight and the concentration of serum total cholesterol (TC), triglyceride (TG) in mice at baseline (4 weeks of age) and the end of the diet treatment period (24 weeks of age).

Age	Group	Body Weight (g)	TC (mmol/L)	TG (mmol/L)
4 weeks	WT	22.3 ± 1.6	3.2 ± 0.6 *	1.4 ± 0.5
	HF+DEF	23.2 ± 0.8	8.9 ± 1.9	1.6 ± 0.5
	HF+CON	23.3 ± 1.6	8.9 ± 1.0	1.6 ± 0.7
	HF+FA	23.0 ± 1.6	9.0 ± 2.7	1.5 ± 0.4
24 weeks	WT	29.9 ± 3.0 *	4.5 ± 2.9 *	2.7 ± 0.6
	HF+DEF	33.4 ± 2.4	15.3 ± 3.2	3.5 ± 2.9
	HF+CON	34.5 ± 2.6	14.7 ± 3.4	3.4 ± 1.5
	HF+FA	37.2 ± 3.9	10.4 ± 2.5 *^,#^	3.1 ± 1.8

Compared with HF+CON group, *, *p* < 0.05; compared with HF+DEF group, #, *p* < 0.05; *n* = 8, mean ± SD.

**Table 2:** Nucleotide sequence of the forward and reverse primers for RT-PCR.

Gene Name	Primer
Mouse-β-actin	Forward: GCTACAGCTTCACCACCACAG Reverse: GGTCTTTACGGATGTCAACGTC
Mouse-*MCP1*	Forward: GCCTGCTGTTCACAGTTGC Reverse: GGTGATCCTCTTGTAGCTCTCC
Mouse-*VEGF*	Forward: CTTGTTCAGAGCGGAGAAAGC Reverse: ACATCTGCAAGTACGTTCGTT
Mouse-*DNMT1*	Forward: CCTAGTTCCGTGGCTACGAGGAGAA Reverse: TCTCTCTCCTCTGCAGCCGACTCA
Mouse-*DNMT3A*	Forward: GGCCGAATTGTGTCTTGGTG Reverse: CCATCTCCGAACCACATGAC
Mouse-*DNMT3B*	Forward: TTCAGTGACCAGTCCTCAGACACGAA Reverse: TCAGAAGGCTGGAGACCTCCCTCTT

## Abbreviations

ApoE *^−^*^/*−*^	Apolipoprotein E-deficient
AS	Atherosclerosis
BSA	Bovine serum albumin
CVDs	Cardiovascular diseases
CHD	Coronary heart disease
DNMTs	DNA methyltransferases
DNMT1	DNA methyltransferase 1
DNMT3A	DNA methyltransferase 3 alpha
DNMT3B	DNA methyltransferase 3 beta
FA	Folic acid
HCY	Homocysteine
HE	Hematoxylin and eosin
HF	High fat diet (fed)
HUVECs	Human umbilical vein endothelial cells
MALDI-TOF	Matrix-assisted laser desorption/ionization time-of-flight
MCP1	Monocyte chemotactic protein-1
OD	Optical density
Ox-LDL	Oxidized low-density lipoprotein
PBS	Phosphate buffered saline
PCR	Polymerase chain reaction
SAH	*S*-adenosylhomocysteine
SAM	*S*-adenosylmethionine
TBST	Tris-buffered saline Tween 20
TC	Total cholesterol
TG	Triglyceride
VEGF	Vascular endothelial growth factor
